# Bile duct stone formation around a Prolene suture after cholangioenterostomy

**DOI:** 10.12669/pjms.321.8985

**Published:** 2016

**Authors:** Qiang Li, Liang Tao, Xingyu Wu, Lingjun Mou, Xitai Sun, Jianxin Zhou

**Affiliations:** 1 Qiang Li, Department of Hepatobiliary Surgery, The Affiliated Drum Tower Hospital of Nanjing University Medical School, Nanjing 210008, Jiangsu Province, China; 2 Liang Tao, Department of Hepatobiliary Surgery, The Affiliated Drum Tower Hospital of Nanjing University Medical School, Nanjing 210008, Jiangsu Province, China; 3 Xingyu Wu, Department of Hepatobiliary Surgery, The Affiliated Drum Tower Hospital of Nanjing University Medical School, Nanjing 210008, Jiangsu Province, China; 4 Lingjun Mou, School of Surgery, The University of Western Australia and Western Australia Liver & Kidney Surgical Transplant Service, Sir Charles Gairdner Hospital, Perth, Western Australia, Australia; 5 Xitai Sun, Department of Hepatobiliary Surgery, The Affiliated Drum Tower Hospital of Nanjing University Medical School, Nanjing 210008, Jiangsu Province, China; 6 Jianxin Zhou, Department of Hepatobiliary Surgery, The Affiliated Drum Tower Hospital of Nanjing University Medical School, Nanjing 210008, Jiangsu Province, China

**Keywords:** Prolene suture, Cholelithiasis, Cholangioenterostomy, Vicryl suture

## Abstract

The iatrogenic cause of bile duct stone formation is mainly due to suture materials, especially silk sutures. In recent years, Prolene and Vicryl sutures have been widely used in biliary surgery, and bile duct stone formation related to sutures are seemingly becoming rare, as there has only been one report of bile duct stone formation caused by Prolene sutures in the literature. In the last few years we have had two cases of Prolene suture-related bile duct stone formation within our unit. We therefore suggest that Vicryl sutures should be used as the first choice in biliary surgery, in order to prevent the formation of iatrogenic bile duct stones.

## INTRODUCTION

The main cause of iatrogenic bile duct stone formation is due to the suture materials, especially silk sutures.^1^ With the development of surgical sutures such as Prolene and Vicryl sutures, which are widely used in biliary surgery, the ratio of suture-related iatrogenic bile duct stone formation appears to have decreased significantly. However, in 2012, Beardsley et al. reported a case of a large stone formation within the biliary tract as a consequence of Prolene.^2^ Here, we present two cases of bile duct stone formation related to the Prolene suture in our department during recent years. As a result of these findings, the safety of Prolene in biliary surgery requiresre-evaluation.

## CASE PRESENTATION

### Case 1

A 37-year-old man was admitted to our department complainingofintermittent episodes of fever over 1.5 months, without abdominal pain, and a highest temperature of 40°C. His past medical history showed that he had suffered bile duct injury as a result of cholecystectomy, and had undergone a Roux-en-Y choledochojejunostomy reconstruction 4 years previously to repair the damage. The bilioenteric anastomosis was fixed with a continuous 3-0 Prolene suture. The following 4 years were uneventful.

**Fig.1:**
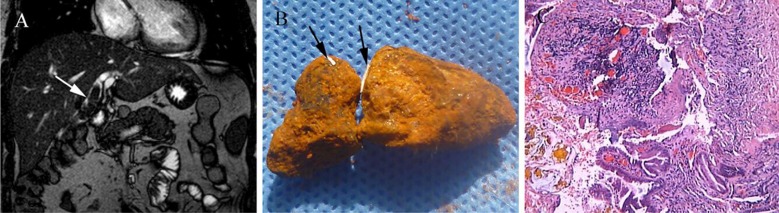
Case 1: A. Magnetic rResonance cholangiopancreatography (T2). The white arrows indicate bile duct stones in the hilum. B. Bile duct stone formed around a Prolene suture. The black arrows indicate the Prolene suture within the stone. C. Pathological examination of the biliary anastomotic tissue.

The patient’s laboratory data revealed the infection and hepatic dysfunction during the current admission: white blood cells, 11.1 × 10^9^/L (4−10×10^9^/L); neutrophils, 86.5% (51−75%); alanine aminotransferase, 159.4 U/L (5−40U/L); aspartate aminotransferase, 50.2 U/L (8−40U/L); alkaline phosphatase, 338.9 U/L (47−185U/L); γ-glutamyltransferase, 612.9 U/L (11−50U/L); direct bilirubin, 11.4μmol/L (1.7−6.8μmol/L). Magnetic resonance cholangiopancreatography (MRCP) showed the common bile duct was indistinguishable, with multiple nodular low signals in the hilar bile duct, and the intrahepatic bile duct was dilated (Fig.1A). This patient underwent a laparotomy, and large full cast stones were found in the common bile duct. The stones were formed around sutures (Fig.1B), which was identified as a Prolene suture under macroscopic examination. After the stones were extracted, the bilioentericanastomotic incision was closed tranversely with an interrupted 4-0 Vicryl suture.

Following surgery, the patient’s laboratory data gradually returned to normal levels before discharge. His postoperative course was uneventful, and choledocholithiasis did not recur after surgery. Pathological examination revealed chronic cholecystitis on mucosal tissue with erosions, hyperplasia of fibrous connective tissue and congestion of the blood vessels in the anastomotic tissue (Fig.1C). The follow up time is 30 months postoperatively, and he was uneventful without any complications, and choledocholithiasis did not recur.

### Case 2

A 72-year-old man was admitted to our department, complaining of a 1-month history of intermittent episodes of right upper quadrant abdominal pain and bloody diarrhoea. His past medical history showed that he had undergone cholecystectomy, choledocholithotomy, and Roux-en-Y choledochojejunostomy reconstruction 5 years previously because of an intrahepatic bile duct stone. Two years following the surgery, he suffered recurrent episodes of cholangitis. Follow-up investigations determined this was due to recurrent bile duct stone formation. He subsequently underwent another choledocholithotomy and bilioenteric anastomotic reconstruction. The following three years were uneventful.

**Fig.2:**
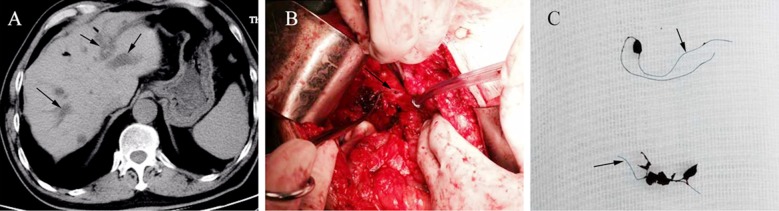
Case 2: A. CT scan, showing dilation of the intrahepatic bile duct (black arrows). B. The stone was found after the anastomosis (black arrow) was opened. C. Several bile duct stonesformed around the Prolene sutures. The black arrows indicate the Prolene sutures.

During the current admission, his laboratory data showed normal liver function with no sign of infection. Colonoscopy and pathological examination showed colon adenocarcinoma. Abdominal ultrasonography identified stones in the bile duct with intrahepatic bile duct dilatation. Computed tomography (CT) showed the intrahepatic bile duct was dilated (Fig.2A). Based on these findings, we concluded that radical laparocolectomy and choledocholithotomy was appropriate for this patient. Following radical laparocolectomy, we opened the bilioenteric anastomosis, and found there were several bile duct stones that had formed around a suture in the enteric end (Fig.2B). On macroscopic examination, the suture was identified as a Prolene suture, which might have been used for overhanging the bile duct wall left in the lumen (Fig.2C). Following the extraction of the stones, the incision at the bilioenteric anastomosis was closed with interrupted 4-0 Vicryl suture transversely. His postoperative course was uneventful without any complications, and choledocholithiasis did not recur after surgery for 18 months follow-up.

## DISCUSSION

There can be a number predisposing factors that can cause stone formation in the biliary duct, such as foreign bodies, benign or malignant strictures, bacterial infection, parasites, metabolic changes and unusual dietary habits.^3^, ^4^ A study conducted by Justin Ban and colleagues at the Harbor General Hospital, Torrance, California and University of California Medical School, Los Angeles, California, USA, reported that non-absorbable suture materials can account for 82% of stones that form around a foreign body^5^ and particularly non-absorbable silk sutures have been found to be responsible for the majority of reported cases of stone formation.^6^ However, the absorbable chromic catgut suture has also been reported to cause calculus formation.

Prolene is a single-strand, smooth suture, which minimises tissue injury. It is resistant to bile erosion and does not cause local inflammation. It can also reduce scar formation and anastomotic stricture development, and has been recommended as an ideal suture for bile duct reconstruction as a non-absorbable suture.^7^ There were no reports about bile duct stone formation related to Prolene sutures until Beardsley et al. reported a case in 2012. In the current study, we also present two further cases of bile duct stone formation associated to this kind of suture. We therefore propose that the safety of the Prolene suture in biliary surgery should be reevaluated.

How do these non-absorbable sutures move into the inner lumen of bile duct and cause bile duct stone formation? Chiyo Maeda and colleagues at the Department of Digestive Surgery, Niigata City General Hospital, Japan suggested that the surrounding tissues can compress the bile duct, resulting in ischemic parietal damage at the site of chronic compression, a process likely to allow the non-absorbable suture materials to migrate into the bile duct.^8^ In addition, Mackie et al demonstrated that non-absorbable suture materials can act as a nidus for the bile stone formation, if they are exposed to the bile duct lumen.^9^

In the present report, the Prolene suture was used for a continuous anastomosis in Case one. Chronic compression of surrounding tissues to the anastomosis might cause ischemica and chronic cholecystitis on mucosal tissue with erosion, and the suture might migrate into the lumen. In Case two, the Prolene suture was left in the lumen during the surgery. Bile duct stone formation had taken place in both cases, primarily due to the non-absorbable sutures floating in the lumen of bile duct. Thus surgical technique which caused the exposure of suture specially the tip or knot of suture to the bile duct lumen may be an important factor for bile duct formation. Although we speculated that the patients might have also been prediclosed to stone formation.

The Vicryl suture is an absorbable, single-strand suture, which has been used in biliary reconstruction. It is not like the traditional absorbable sutures such as chromic catgut. Vicryl sutures are absorbed within 60 to 90 days through hydrolysis and enzymatic degradation. A usual reaction involves a minimal multicellular inflammatory infiltrate with neutrophils, eosinophils, fibroblasts, microphages and giant cells.^10^ It is able to have little impact on surrounding tissues, and does not cause scar formation. It could preserve excellent tensile strength after soaking in the bile in vitro.^11^ In these two cases, we closed the bilioenteric anastomoses by using interrupted 4-0 Vicryl sutures transversely. The two patients’ postoperative courses were both uneventful, without choledocholithiasis recurrence in the follow-up evaluation. We believe that Vicryl may be absorbable, without causing bile duct stone formation.

Prolene has been widely used in biliary surgery for several years, and stone formation related to this type of suture is relatively uncommon. It remains to be determined whether the Prolene suture could cause bile duct stone formation when it is exposed to the bile duct lumen. The safety of prolene for cholangioenterostomy is debatable and needs to be re-visited. As an absorbable suture which could tolerate soaking in bile, and no negative reports for its association with iatrogenic bile duct stone formation, we therefore propose that Vicryl could be considerable for biliary surgery.
